# Clinicians’ experience on the effectiveness of pharmacotherapy in patients with first-episode depression: a randomized controlled trial based on pharmacogenomics

**DOI:** 10.3389/fphar.2025.1626654

**Published:** 2025-08-07

**Authors:** Jie Tong, Jie Yuan, Yu Qin, Na Zhu, Tingting Zhang, Xiaochun Zhu, Yuanyuan Xu, Meilin Liu, Jie Zhang, Xirong Sun

**Affiliations:** Clinical Research Center for Mental Disorders, Shanghai Pudong New Area Mental Health Center, School of Medicine, Tongji University, Shanghai, China

**Keywords:** psychiatrist, first-episode depression, depression, FED, pharmacogenomics, PGx

## Abstract

**Background:**

Due to differences in drug efficacy, the risk of adverse reactions, and individual differences between patients, clinicians face significant challenges in terms of selecting drugs for the treatment of depression. However, no previous studies have compared the efficacy of antidepressant treatments between psychiatrists with different levels of experience based on pharmacogenomics approach.

**Methods:**

A total of 178 patients were recruited and randomly assigned to pharmacogenomics-guided treatment group or regular treatment control group. The control group was further divided into the senior doctor and the nonsenior doctor subgroups. All participants completed pharmacogenomic assessments at baseline. They also completed the 17-items Hamilton Depression Scale (HAM-D_17_), Dimensional Anhedonia Rating Scale (DARS), Perceived Deficits Questionnaire-Depression (PDQ-D), and Antidepressant Side Effect Checklist (ASEC) at baseline and at 4-week, 8-week, 16-week, and 32-week follow-ups. The study protocol was registered with *ClinicalTrials.gov* (NCT05669391).

**Results:**

Compared with the control group, pharmacogenomics-guided group presented significant differences in DARS and ASEC scores at 32 weeks (*P*_Bonferroni_< 0.05), with significant time and group effect (*P* < 0.05). However, there was no significant difference in HAM-D_17_ and PDQ-D scores between the two groups at 32 weeks (*P*_Bonferroni_> 0.05). The number of antidepressant changes at 32 weeks in the nonsenior doctor subgroup was significantly higher than that in the senior doctor subgroup (1.04 vs. 0.31, *P* = 0.005). There was no significant difference in the number of combined antidepressants, the number of patients who used somnifacients, HAM-D_17_ scores, DARS scores, PDQ-D scores, and ASEC scores between the two groups (*P* > 0.05). The number of antidepressant changes and HAM-D_17_ scores are 32-week were negatively correlated with the doctor’s years of work experience (r = −0.25, *P* = 0.012; r = −0.29, *P* = 0.004; respectively).

**Conclusion:**

Pharmacogenomics-guided treatment can effectively mitigate the occurrence of anhedonia and side effects in patients with first-episode depression. Higher level of clinical experience among psychiatrists can lead to significant reduction in the frequency of antidepressant drug changes, and the depressive symptoms at the endpoint are negatively correlated with the clinicians’ work experience. Pharmacogenomics may reduce the influence of clinical experience on treatment outcomes in primary mental healthcare settings.

## Background

Depression is an affective disorder that is characterized by sustained low mood, loss of interest, and even suicidal ideation and behaviour (Mangione et al., 2022). Depression has a high prevalence, recurrence rate, and disease burden. Currently, the standard treatment for depression include medication, psychological therapy, and neural regulation; at least 50% of patients with depression are treated with antidepressant medication (Zaidi et al., 2024). According to data from the National Health Service (NHS) in the United Kingdom, over 70 million prescriptions for antidepressants are dispensed each year, and the total cost of antidepressants worldwide is expected to reach $17.6 billion by 2030 (James and John, 2018). A survey by the Centers for Disease Control and Prevention in the United States revealed that antidepressants have become the most widely used prescription drugs in the country, with prescription volumes even increasing by 18.6% in the first quarter of 2020 (Haddad et al., 2021).

Currently, there are six types of antidepressants worldwide, encompassing nearly 70 drugs; furthermore, more than 240 antidepressants are currently in the clinical Phase I New Drug Application (NDA) stage (Skovlund et al., 2016). Clinicians may face significant challenges in choosing antidepressants based on the drug efficacy, adverse reactions, and individual differences among patients (Read et al., 2023). A previous study revealed that only 30%–45% of patients with depression achieve clinical remission with a single antidepressant treatment in clinical practice, and another 10% of patients reported that any type of antidepressant treatment was ineffective (Mekonen et al., 2021). A study conducted in the primary healthcare setting showed that patients with depression switch drugs an average of three times (Phelps and James, 2017). Even the meta-analysis study revealed that the use of fluoxetine and venlafaxine in adolescents may lead to a significantly higher risk of suicide than in adults (Gibbons et al., 2012). Notably, the National Institute of Mental Health’s Sequenced Treatment Alternatives of Relieve Depression (STAR*D) trial revealed that patients taking medication for first-episode depression have a remission rate of less than 50% when using Quick Inventory of Depressive Symptomatology-Self-Report (QIDS-SR) as an evaluation metric (Sinyor et al., 2010). In clinical practice, newly trained physicians may only focus on the outcome of depressive symptoms and easily overlook other influencing factors of the subjects. This may lead to differences in the onset time of symptoms in different dimensions and the risk of adverse drug events. Therefore, patients hope to obtain better clinical efficacy or reduce the risk of antidepressant treatment by visiting highly qualified or experienced clinicians or adhering to precision medicine techniques (McIntyre et al., 2023).

The use of pharmacogenomics in the precision treatment of depression is receiving increased attention (Kalin, 2024). By directly detecting gene sequences, the correlation between gene sequence differences and antidepressant drug effects can be verified (Winner et al., 2013). Most pharmacogenomic tests focus on gene variations encoding hepatic cytochrome P450 enzymes, as well as 5-HT transporter (5-HTT) and brain-derived neurotrophic factor (BDNF); furthermore, some pharmacogenomic tests focus on gene variations encoding FKBP5 protein and pregnane X receptor (PXR), which are regulated by the hypothalamic-pituitary-adrenal (HPA) axis (Juanjuan et al., 2020). Aldrich et al. (2019) reported that depressed patients with a high metabolism of CYP2C19 were more likely to experience adverse reactions, including agitation and weight gain, after using citalopram. A prospective study by Gassó et al. (2017) revealed that variations in the HTR1B gene and methylation levels in the promoter gene region of TFBS were associated with clinical improvement among adolescents with depression who were treated with fluoxetine. Overall, pharmacogenomic testing can improve drug selection or dosage in patients with genetic variations that alter pharmacokinetics or pharmacodynamics, thereby increasing drug efficacy and safety and providing guidance for rational clinical application.

However, there are inconsistent findings in clinical evidence-based research ov antidepressant pharmacogenomics. Greden et al. (2019) suggest that pharmacogenomic testing may be particularly helpful in treating major depressive disorder (MDD), with 28%–33% of MDD patients experiencing reduced response and treatment participation rates in each treatment trial. A short-term randomized study revealed that patients who underwent pharmacogenomic testing for a set of pharmacokinetic- and pharmacodynamic-related variants did not show significant improvement in primary efficacy outcomes at the 8-week follow-up assessment (Perlis et al., 2020). A real-world cohort study showed that PGx guided antidepressant medication treatment for depression patients aged 15-24 reduced suicide related emergency visits by 52% (Ofoegbu and Ettienne, 2014). Psychiatrists havelong on subjective characteristics such as patient examination, self-report, questionnaire surveys, and personal clinical experience, which may lead to differences in the efficacy of antidepressant treatment between clinicians with with different levels of experience (Sharaev et al., 2022). Patients often believe that senior clinicians have an advantage in terms of treatment effectiveness. Currently, there is no tools to predict the efficacy evaluation of clinicians with different levels of experiences.

The Primary Mental Healthcare (PMHC) studies focus on multidimensional and long-term evidence in standardized clinical practice for patients with depression. The current prospective, randomized controlled trial aimed to test two main research hypotheses: (1) Do clinicians select fewer antidepressants with potential drug-gene interactions on the basis of pharmacogenomics test results, which can have a long-term impact on symptom relief?; (2) Do highly qualified or experienced psychiatrists have an advantage in terms of treatment efficacy or adverse reactions of antidepressant medication among patients with first-episode depression?

## Methods

### Study design

The randomized controlled trial was conducted at the Clinical Research Center of Shanghai Pudong Mental Health Center, School of Medicine, Tongji University. The sample size was calculated using sample size power analysis software (PASS version 21.0.3 [NCSS LLC, Utah, United States]), with a significance level of 0.05 and a power value of 0.8 for a two-sided test. The participants were divided into two groups; the pharmacogenetic group and the control group. At least 72 participants were assigned to each arm. Assuming a potential 10% dropout rate, the final sample size was determined to be 80 people in each group. The study protocol was registered with *ClinicalTrials.gov* (NCT05669391).

## Participants

All participants were recruited from the Shanghai Pudong Mental Health Center, School of Medicine, Tongji University, from 1 January 2023, to 31 January 2024. The inclusion criteria were as follows: (1) met the diagnostic criteria for depression in the fifth edition of the American Diagnostic and Statistical Manual of Mental Disorders (DSM-5); (2) aged 18–65 years; (3) had first-episode depression and had never used antidepressants; (4) had two biological parents who were Chinese nationals; (5) had visual and auditory discrimination and no obstacle to understanding instructions; (6) were able to cooperate with treatment and complete assessments independently; (7) had an education level above primary school; and (8) provided written informed consent. If the patient was incapacitated during the onset of the disease, written informed consent was obtained from the legal guardian. The exclusion criteria were as follows: (1) met the DSM-5 diagnostic criteria for schizophrenia, schizoaffective disorder, bipolar affective disorder, mental retardation, generalized developmental disorder, delirium, dementia, cognitive dysfunction, alcohol dependence, or other diagnosis; (2) had serious organic diseases, such as diabetes, thyroid disease, hypertension, cardiovascular disease, brain injury, and cerebral ischaemia or haemorrhage; (3) had narrow angle glaucoma; (4) had a history of epilepsy or febrile convulsion; (5) had taken drugs in the past; (6) were positive for syphilis-specific antibodies and AIDS antibodies; (7) had undergone modified electroconvulsive therapy (MECT), repetitive transcranial magnetic stimulation (rTMS) or other neuromodulation therapy before enrolment; (8) had a risk assessment indicating a serious suicide attempt or excitement; (9) had a laboratory examination indicating impaired liver and renal function; (10) were pregnant or lactating or planned to become pregnant soon; and (11) had other contraindications to antidepressants.

### Randomization

The subjects were assigned a number on the basis of the time of enrolment. A researcher who did not participate in the assessments randomized the numbers through SPSS 26.0 software (SPSS, Inc., Chicago, IL, United States) and divided the patients into the pharmacogenetic group and the control group at a ratio of 1:1. After the subjects were allocated to groups, they were assigned a ranking number according to their enrolment time. Finally, the participants were assigned to the corresponding groups according to the results of previous random sampling. The participants were not aware of the grouping allocation.

### Pharmacogenomics (PGx)

The pharmacogenomic approach used in this study involved the genomic analysis of genes associated with 23 antidepressants that are commonly used in clinical practice, including 12 drug metabolism genes and 55 gene detection sites. Tris-EDTA anticoagulation and salt precipitation methods were used to extract DNA, with OD 260/280 values ranging from 1.6 to 1.8 and concentration greater than 50 ng/μL. The PCR gene chip detection method and Illumina Asian Screening Array (ASA) chip detection technology (the first genome-wide single-nucleotide polymorphism (SNP) chip designed using genome-wide sequencing data of 9000+ East Asian individuals, containing 750,000 markers and 85% effective loci) were used to obtain genetic information related to individual differences in the effects of drugs between subjects. The selection of gene loci was based on the International Genealogy Pharmacology Database (PharmGKB), antidepressant drug guidelines, drug instructions, Chinese population frequency, and clinical trial big data customized for antidepressant drug gene testing panels. Suitable antidepressant drugs with pharmacokinetics and pharmacodynamics data were selected for patients.

The experimenter collects a dry blood sample from the end of the subject’s finger. Approximately 50 μL of whole blood was added to a sterile filter paper printing ring and allowed to dry naturally at room temperature for at least 4 h. The blood spots were not stacked during collection and did not contact other interfaces. After the blood spots had completely dried, they were placed in sterile bags, and desiccants and humidity indicator cards were added. After the packaging was sealed, the sample was sent to the laboratory for testing within 8 h.

## Assessments

### 17-Items Hamilton Depression Scale (HAM-D_17_)

The HAM-D_17_ consists of 17 items that assess the severity of a patient’s depressive state and treatment effectiveness, typically serving as a standard for alleviating depressive symptoms. Whisman et al. published 17-items HAM-D scale, and Zhang et al. created a localized Chinese version. The Cronbach α coefficient was 0.77, and the test-retest reliability was 0.95 (Sun et al., 2017). A score less than 7 indicates no depressive symptoms; 7-13 indicates mild depression; a score of 14–19 indicates moderate depression; and a score ≥20 indicates severe depression or above.

## Dimensional Anhedonia Rating Scale (DARS)

The DARS includes 17 items that assess pleasurable activities or experiences among depressed patients across four dimensions (Rizvi et al., 2015). The DARS was developed by Sakina et al., in 2015, and Jia et al. created a localized Chinese version. The Cronbach α coefficient was 0.97, and the test-retest reliability was 0.89 (Hailing et al., 2020). Higher scores indicate a lower degree of happiness deficiency.

### Perceived Deficits Questionnaire-Depression (PDQ-D)

The PDQ-D includes 20 items that assess subjective cognitive dysfunction in people with depression within the past week. It includes 4 subscale (attention/concentratio, retrospective memory, prospective memory, and planning/organization) (Sullivan et al., 1990). The PDQ-D was originally developed by Dr. Michael Sullivan, and Chuan et al. created a localized Chinese version. The Cronbach α coefficient was 0.95, and the test-retest reliability was 0.84 (Shi et al., 2017). Higher score indicate more severe subjective cognitive impairment.

### Antidepressant Side Effect Checklist (ASEC)

The ASEC was developed by the Royal College of the Psychiatrist, was used to classify the reported side effects associated with antidepressant drugs into mild, moderate, and severe. The ASEC assess 21symptoms, such as physical fatigue, dizziness, headache, sleep disorders, orthostatic collapse, palpitations, tremors, sweating, dry mouth, constipation, urinary system disorders, drowsiness, and sexual dysfunction. The Cronbach α coefficient was 0.78, and the test-retest reliability was 0.64 (Uher et al., 2009). Higher scores indicate more severe side effects.

### Procedure

After randomization, all participants completed pharmacogenomic sampling at baseline. The participants also underwent psychopathological evaluation at baseline and at the 4-week, 8-week, 16-week, and 32-week follow-ups. Under the experimenter’s guidance, the participants completed the assessment in a quiet room; the experimenter checked the completion status of each project. The evaluator holds a master’s degree in psychiatry and is registered as a clinical psychological scale surveyor in China. In the pharmacogenomics guidance group, clinically practising psychopharmacology pharmacist classify antidepressants into low-risk, medium risk, and high-risk categories based on the pharmacogenomics test results of subjects. Clinicians ultimately selected antidepressants with with a lower risk of drug‒gene interactions risk for subjects. In the conventional treatment control group, clinicians were directed to provide treatment as usual. All expenses related to pharmacogenomics were covered by this study.

### Blinding strategy

To maintain research blinding, all the subjects in this study underwent pharmacogenomic sampling, but only the intervention group underwent pharmacogenomic testing. To reduce the variability of data collection and potential unblinding, only one clinically practising psychopharmacology pharmacist interpreted the pharmacogenomic results, and only one trained assessor administered the assessments. The subjects, psychopharmacology pharmacists, and psychological evaluator were blinded to the group allocation. The study grouping and pharmacogenomic testing results were provided to the subjects after the end of the study.

### Statistical analysis

Data analysis was conducted via SAS software (version 9.4 SAS Institute, Inc.). The distribution of the data was determined via the Kolmogorov-Smirnov one-sample test. The qualitative data are presented as percentages, and the quantitative data are expressed as the mean ± standard deviation (SDs). The baseline demographic and clinical characteristics were compared via the chi-square test and analysis of variance (ANOVA). The intent-to-treat (ITT) analysis was used for sensitivity analysis, and the last-observation-carrying-forward (LOCF) principle was used to replace missing data.

Between-group factors (pharmacogenomic-guided group and regular control group) and within-group factors (baseline, week 4, week 8, week 16, and week 32) were assessed after adjustment for confounding covariates. Independent sample t tests were used for comparisons between two groups. Repeated-measures multivariate analysis of variance (RM MANOVA) was used to obtain each score and the overall *P* value of HAM-D_17_, DARS, PDQ-D, and ASEC. Repeated-measure analysis of variance (RM ANOVA) was used to examine the scores of the HAM-D_17_, DARS, PDQ-D, and ASEC, thus establishing intergroup and intragroup factors while adjusting for confounding covariates. A follow-up multivariate omnibus test was performed, and each univariate effect was detected via analysis of covariance (ANCOVA). If the group × time interaction was significant, ANCOVA was used to analyse the group differences in the within-group factors. Independent sample t tests or RM MANOVA were used to compare the evaluation indicators for various dimensions of the study endpoint between the senior doctor subgroup and the non-senior doctor subgroup within the control group. Bonferroni corrections were applied to correct for multiple tests. Pearson’s correlation analysis was used to evaluate the correlation between doctors’ work experience and medication use and symptom outcomes. The power and sample size calculation were performed based on a two-tailed *P*-value of 5%. Effect sizes are described as Cohen’s d. Differences were considered statistically significant at *P* < 0.05.

## Ethics

The protocol for this study was approved by the Research Ethics Committee of the Shanghai Pudong New Area Mental Health Center and Tongji University School of Medicine (No: PDJWLL2022033). All procedures were performed in accordance with the ethical standards of the responsible committees on human experimentation (institutional and national) and the Helsinki Declaration of 1975, as revised in 2024. The participants provided written informed consent for participation in this study.

## Results

### Clinicians

A total of 31 clinicians consented to participate in the study. While the protocol capped the number of randomized patients per clinician at 10, the distribution of the number of randomizations per clinician varied. Among clinicians who had at least 1 randomized patient, those with greater time in clinical practice, those who practiced in a mental health clinic, and those who had advanced professional certificates had more randomized patients. Among the 178 participants, 51.69% had clinicians with seniority or clinicians who were above technical level, with an average work experience of 18.25 ± 4.98 years. The number of antidepressants changes was 0.67, and the number of types of combined antidepressants was 1.10. A total of 51.68% of the clinicians prescribed hypnotic drugs in combination (Table 1).

**TABLE 1:** Participant demographics and social characteristics.

Characteristic	Total (n = 178)	PG (n = 81)	CG (n = 97)	t/χ^2^	*P* value^a^
Age, Mean (SD). y	34.34 (15.53)	33.48 (16.40)	35.05 (14.83)	−0.77^a^	0.444
Sex, No. (%)					
Male	56 (31.46)	26 (14.61)	30 (16.85)	0.03^b^	0.867
Famale	122 (68.54)	55 (30.90)	67 (37.64)		
Years of education, Mean (SD). y	13.79 (3.02)	13.68 (3.01)	13.89 (3.04)	−0.46^a^	0.649
Marital status, No. (%)					
Single	95 (53.37)	42 (23.60)	53 (29.78)	5.38^b^	0.146
Married	74 (41.57)	37 (20.79)	37 (20.79)		
Divorced or widowhood	9 (5.06)	2 (1.12)	7 (3.93)		
Employment, No. (%)					
Employed	95 (53.37)	40 (22.47)	55 (30.90)	0.98^b^	0.614
Unemployed	56 (31.46)	28 (15.73)	28 (15.73)		
Retire	27 (15.17)	13 (7.30)	14 (7.87)		
Course of disease, Mean (SD). m	26.51 (39.81)	27.41 (45.17)	25.76 (34.95)	0.27^a^	0.785
BMI, Mean (SD), kg/m^2^	21.92 (3.10)	21.63 (3.44)	21.17 (2.78)	−1.16^a^	0.248
Doctor’s technical level, No. (%)					
Junior and below	86 (48.31)	34 (41.97)	52 (53.60)	6.12^b^	0.153
Senior and above	92 (51.69)	47 (58.03)	45 (46.40)		
Doctor’s years of work experience, Mean (SD). y	18.25 (4.98)	18.74 (4.75)	17.91 (4.49)	4.52^a^	0.064
Number of antidepressant changes, Mean (SD). y	0.67 (0.83)	0.35 (0.64)	0.94 (0.88)	−5.07^a^	<0.001^e^
Number of combined antidepressants, Mean (SD). y	1.10 (0.30)	1.11 (0.32)	1.09 (0.29)	0.40^a^	0.688
Using somnifacient, No. (%)					
Yes	92 (51.68)	40 (49.38)	52 (53.60)	0.32^b^	0.574
No	86 (48.42)	41 (50.62)	45 (46.40)		

Abbreviations: PG, pharmacogenomic-guided group; CG, control group; BMI, body mass index.

^a^ Two-sample t-test.

^b^ Chi-squared test.

^c^ *P* < .05.

^d^ *P* < .01.

^e^ *P* < .001.

### Patients

**FIGURE 1:**
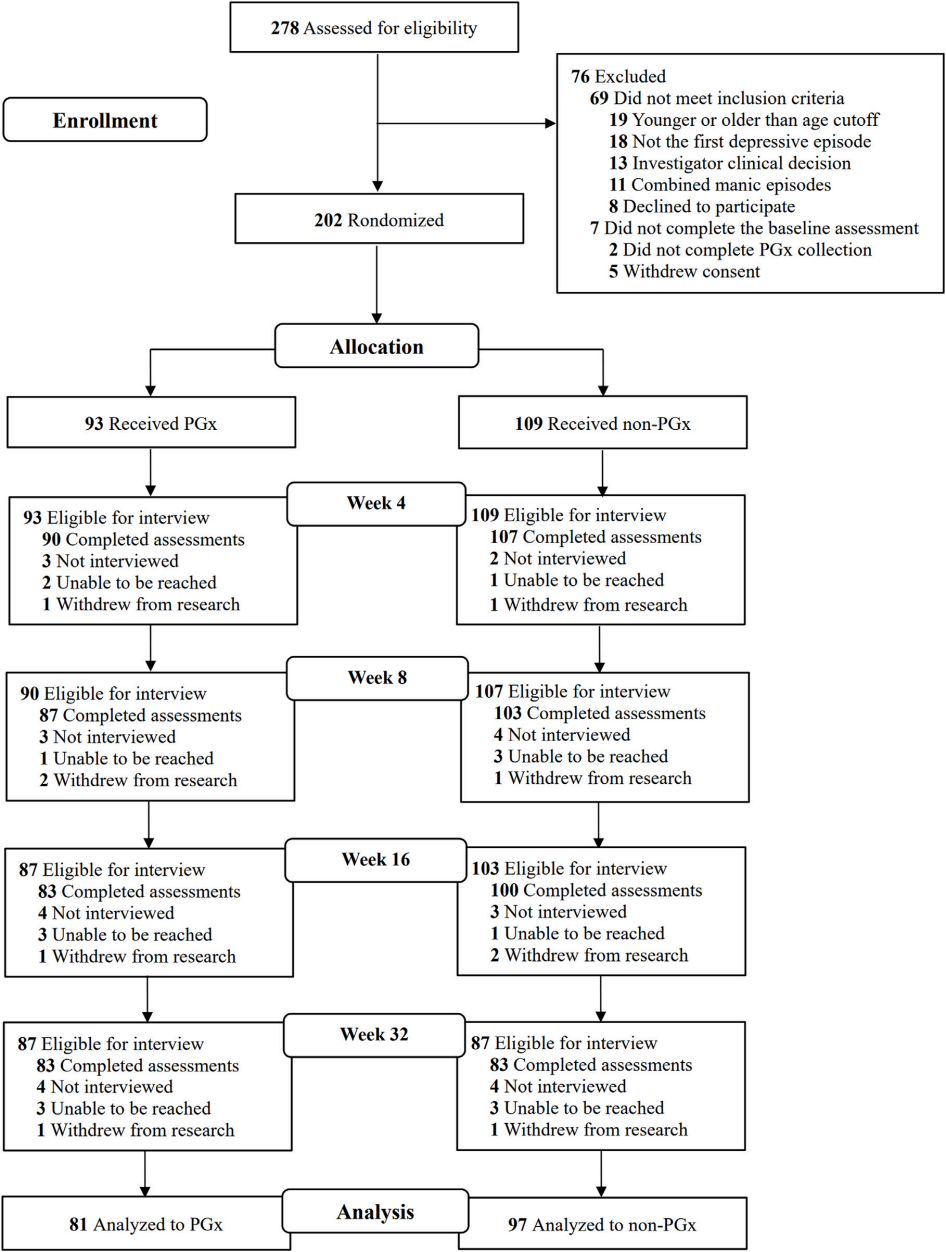
Screening, enrollment, randomization, and outcomes among individuals in the study. PGx: Pharmacogenomics.

A total of 278 patients consented to participate, of whom 202 were randomized (Figure 1). The demographic characteristics, including age, sex, education, marital status, employment status, disease course, and body mass index (BMI), of the participants in the pharmacogenomic-guided, control, and full sample groups are shown in Table 1. Among the 178 participants, the average age was 34.34 ± 15.53 years, and the proportion of females was 68.54%. The average number of years of education was 21.92 ± 3.10, and most participants were married (41.57%). A total of 53.37% of the participants were employed, and the average course of disease was 26.51 ± 39.81 months. The average BMI was 21.92 ± 3.10. There were no significant differences in the demographic characteristics between the pharmacogenomic-guided group and control group (*P* > 0.05).

### Depressive symptom outcomes

**FIGURE 2:**
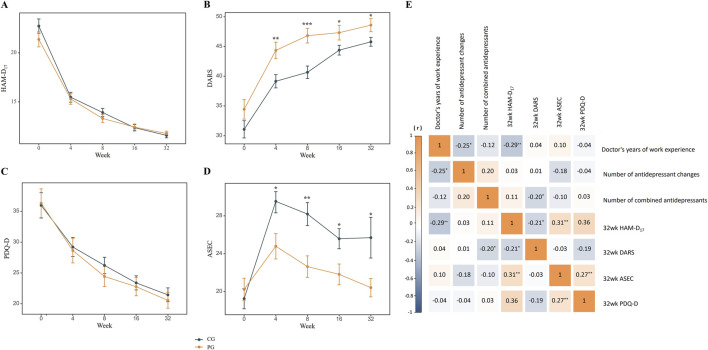
The impact of pharmacogenomics on various dimensions of first-episode depression and correlation analysis within the control group. **(A)**, No significant difference in HAM-D_17_ score; **(B)**, Improvement in DARS score; **(C)**, No significant difference in PDQ-D score; **(D)**, Improvement in ASEC score; **(E)**, Correlation analysis of remission in various dimensions of study endpoints within the control group. Abbreviations: HAM-D_17_: 17-items Hamilton Depression Scale; DARS: Dimensional Anhedonia Rating Scale; PDQ-D: Perceived Deficits Questionnaire-Depression; ASEC: Antidepressant Side Effect Checklist; PG, pharmacogenomic-guided group; CG, control group. ^*^ *p* < 0.05, ^**^ *p* < 0.01, ^***^ *p* < 0.001.

As shown in Table 2 and Figure 2, HAM-D_17_, DARS, and PDQ-D scores were used to measure the alleviation of depressive symptoms in patients receiving antidepressant prescriptions. There was no significant difference in HAM-D_17_ scores between the pharmacogenomic-guided group and control group at any time points within 32 weeks (*P* > 0.05). RM MANOVA was conducted using HAM-D_17_ score as the outcome measure and BMI as the covariate; the results revealed a significant time effect (Wilks’ lambda *F* = 387.61, *P* < 0.001, η^2^ = 0.69), whereas the group effect and group × time interaction were not significant (Wilks’ lambda *F* = 0.45, *P* = 0.504, η^2^ = 0.01; Wilks’ lambda *F* = 2.20, *P* = 0.127, η^2^ = 0.12; respectively). After adjusting for BMI, age, sex, disease course, and baseline DARS scores, the pharmacogenomic-guided group exhibited significant differences in DARS scores at weeks 4, 8, 16, and 32 compared with those of the control group (*P*_Bonferroni_< 0.05, Cohen’s d = 0.23–0.58). RM MANOVA was also conducted with the DARS score as the outcome measure and BMI as the covariate; the results revealed significant time and group effects (Wilks’ lambda *F* = 165.25, *P* < 0.001, η^2^ = 0.48; Wilks’ lambda *F* = 7.59, *P* = 0.007, η^2^ = 0.41; respectively), whereas the group × time interaction was not significant (Wilks’ lambda *F* = 2.85, *P* = 0.070, η^2^ = 0.16). The PDQ-D scores decreased in the pharmacogenomic guidance group, but the mean differences between the pharmacogenomic guidance group and the control group at each time point up to 32 weeks were not significant (*P*_Bonferroni_> 0.05). RM MANOVA was conducted with PDQ-D scores as the outcome measure and BMI as the covariate; the results revealed a significant time effect (Wilks’ lambda *F* = 188.61, *P* < 0.001, η^2^ = 0.52), whereas group and the group × time interaction effects were not significant (*P* > 0.05).

**TABLE 2:** Effect of pharmacogenomic-guided group vs. regular control group on depressive symptoms and adverse reactions.

Time point	Group, Mean (SD)		Estimated group effect within time point			Time effect			Group effect			Time × Group interaction		
	PG	CG	Difference^a^ (95% CI)	*P* value	*d* ^b^	F_1.40_	*P* value	η^2^	F_1.40_	*P* value	η^2^	F_1.40_	*P* value	η^2^
HAM-D_17_														
BL	21.37 (6.89)	22.73 (7.18)	−1.36 (−3.46 to 0.73)	0.201	0.19	387.61	<0.001^c^	0.69	0.45	0.504	0.01	2.20	0.127	0.12
4weeks	15.30 (4.82)	15.47 (4.85)	−0.18 (−1.61 to −1.26)	0.807	0.04									
8weeks	13.31 (3.51)	13.92 (4.11)	−0.61 (−1.75 to 0.53)	0.295	0.16									
16weeks	12.43 (2.81)	12.37 (3.25)	0.06 (−0.85–0.97)	0.895	0.02									
32weeks	11.78 (2.13)	11.59 (2.48)	0.19 (−0.50–0.88)	0.588	0.08									
DARS														
BL	34.44 (14.79)	31.07 (14.18)	3.37 (−0.92–7.67)	0.123	0.23	165.25	<0.001^c^	0.48	7.59	0.007^d^	0.41	2.85	0.070	0.16
4weeks	44.35 (12.49)	39.15 (11.05)	5.19 (1.71–8.67)	0.004^d^	0.44									
8weeks	46.81 (11.04)	40.66 (10.34)	6.16 (2.99–9.32)	0.000^c^	0.58									
16weeks	47.32 (11.08)	44.38 (8.06)	2.94 (0.11–5.78)	0.042^e^	0.31									
32weeks	48.59 (10.01)	45.78 (7.74)	2.81 (0.23–5.38)	0.032^e^	0.32									
PDQ-D														
BL	36.40 (21.16)	35.97 (20.32)	0.34 (−5.79–6.47)	0.913	0.02	188.61	<0.001^c^	0.52	0.11	0.743	0.01	0.78	0.427	0.01
4weeks	28.63 (17.92)	29.21 (15.14)	−0.58 (−5.46 to 4.31)	0.812	0.04									
8weeks	24.43 (14.78)	26.23 (13.05)	−1.80 (−5.91 to 2.32)	0.391	0.13									
16weeks	22.78 (13.20)	22.39 (11.25)	−0.61 (−0.85–0.97)	0.895	0.05									
32weeks	20.56 (11.45)	21.45 (11.03)	−0.90 (−4.23 to 2.44)	0.596	0.08									
ASEC														
BL	20.22 (10.67)	19.25 (10.53)	0.98 (−2.17–4.13)	0.542	0.09	21.61	<0.001^c^	0.11	5.71	0.018^e^	0.03	5.04	0.009^d^	0.03
4weeks	24.79 (12.12)	29.53 (12.54)	−4.74 (−8.41 to −1.17)	0.012^e^	0.38									
8weeks	22.63 (10.23)	28.19 (11.97)	−5.56 (−8.89 to −2.23)	0.001^d^	0.50									
16weeks	21.81 (9.86)	25.58 (10.48)	−3.76 (−6.79 to −0.73)	0.015^e^	0.37									
32weeks	20.41 (8.74)	25.69 (21.19)	−5.28 (−10.25 to −0.32)	0.037^e^	0.32									

Abbreviations: HAM-D_17_: 17-items Hamilton Depression Scale; DARS: dimensional anhedonia rating scale; PDQ-D: Perceived Deficits Questionnaire-Depression; ASEC: antidepressant side effect checklist; PG, pharmacogenomic-guided group; CG, control group; BL, baseline; wk, week.

^a^ Differences between pharmacogenomic-guided group and control group.

^b^ Effect sizes are listed as Cohen`s *d*.

^e^ *P* < .05.

^d^ *P* < .01.

^c^ *P* < .001.

### Analysis between clinicians and endpoint outcomes

As shown in Table 3, the number of antidepressant changes in the nonsenior doctor subgroup was significantly higher than that in the senior doctor subgroup at 32 weeks endpoint (1.04 vs. 0.31, *P* = 0.005). There was no significant differences in the number of combined antidepressants, the number of patients who used somnifacients, the HAM-D_17_ scores, DARS scores, PDQ-D scores, and ASEC scores between the two groups at the 32-week endpoint (*P* > 0.05). Pearson’s correlation analysis was conducted to determine the associations between doctors’ work experience, medication use, and the symptom assessment outcomes at the study endpoints. The number of antidepressant changes and HAM-D_17_ at 32 weeks were significantly and negatively correlated with the doctor’s years of work experience (r = −0.25, *P* = 0.012; r = −0.29, *P* = 0.004; respectively). The number of combined antidepressants and 32-week HAM-D_17_ scores were significantly and negatively correlated with the 32-week DARS scores (r = −0.20, *P* = 0.046; r = −0.21, *P* = 0.034; respectively). The 32-week HAM-D_17_ and 32-week PDQ-D scores were significantly and positively correlated with the 32-week ASEC scores (r = 0.31, *P* = 0.002; r = 0.27, *P* = 0.008; respectively). There was no significant relationship between any other variables (*p* > 0.05) (Figure 2E).

**TABLE 3:** Comparison of medication and remission outcomes among different experienced doctors in the control group at the study endpoint.

Characteristic	nSG (n = 52)	SG (n = 45)	*t* / χ^2^	*P* value^a^
Number of antidepressant changes, Mean (SD). y	1.04 (0.88)	0.31 (0.48)	2.89^a^	0.005^d^
Number of combined antidepressants, Mean (SD). y	1.10 (0.29)	1.08 (0.28)	0.21^a^	0.834
Number of using somnifacient, No. (%)	0.52 (0.51)	0.62 (0.50)	−0.61^b^	0.543
32weeks HAM-D_17_, Mean (SD). y	11.69 (2.57)	10.92 (1.70)	1.04^a^	0.301
32weeks DARS, Mean (SD). y	45.46 (7.51)	47.85 (5.95)	−1.09^a^	0.278
32weeks PDQ-Q, Mean (SD). y	21.61 (11.20)	20.46 (10.28)	0.35^a^	0.729
32weeks ASEC, Mean (SD). y	25.67 (22.49)	25.85 (9.78)	−0.03^a^	0.978

Abbreviations: nSG, nonsenior doctor subgroup; SG, senior doctor subgroup; HAM-D_17_: 17-items Hamilton Depression Scale; DARS: dimensional anhedonia rating scale; PDQ-D: Perceived Deficits Questionnaire-Depression; ASEC: antidepressant side effect checklist; wk, week.

^a^ Two-sample t-test.

^b^ Chi-squared test.

^c^ *P* < .05.

^d^ *P* < .01.

^e^ *P* < .001.

### Side effects and safety

After adjusting for BMI, age, sex, disease course, and baseline ASEC scores, the pharmacogenomic-guided group presented significant differences in ASEC scores at weeks 4, 8, 16, and 32 compared with the control group (*P*_Bonferroni_< 0.05, Cohen’s d = 0.32–0.50). Moreover, after adjusting for BMI as a covariate, RM ANOVA revealed signifcant time, group, and group × time interaction effects on the ASEC score (Wilks’ lambda *F* = 21.61, *P* < 0.001, η^2^ = 0.11; Wilks’ lambda *F* = 5.71, *P* = 0.018, η^2^ = 0.03; Wilks’ lambda *F* = 5.04, *P =* 0.009, η^2^ = 0.03; respectively). Additionally, no adverse events were found to be related to the intervention (Table 2; Figure 2D).

## Discussion

To our knowledge, this is the first study to explore the differences in HAM-D_17_, DARS, PDQ-D, and ASEC scores in patients with first-episode depression and the correlation of these scores with clinicians’ level of experience in a prospective, long-term, randomized controlled trials guided by pharmacogenomics. The main findings of this study were as follows: (i) Compared with the non-pharmacogenomics guided group, antidepressant treatment based on pharmacogenomics led to significant differences in the impacts of anhedonia and drug side effects on patients both within and between two groups at different time points. (ii) The number of antidepressant changes in the nonsenior doctor subgroup was significantly higher than that in the senior doctor subgroup at the endpoint, but there were no significant differences in the number of combined antidepressants used, the number of patients using somnifacient, depressive symptoms, and adverse drug reactions. (iii) The number of antidepressant changes and the depressive symptoms at the endpoint were significantly and negatively correlated with doctor’s years of work experience.

Due to limitations in research methods, previous studies have reported inconsistent results regarding the use of pharmacogenomics as a clinical support tool to improve treatment outcomes in patients with depression. Roy et al. (Perlis et al., 2020) performed a multicenter randomized controlled trial and found no significant difference in the remission rate (measured via the HAM-D_17_) between the pharmacogenomics-guided group and the control group in patients with MDD. Michael et al. (McCarthy et al., 2021) used the Clinical Global Impression (CGI) scale and the QIDS-SR as outcome measures and reported that the pharmacogenomic group improved faster than the control group; however, the difference was not statistically significant. Nonetheless, a real-world randomized controlled study revealed that the treatment response rate of in MDD patients in the pharmacogenomic group was significantly faster than that in patients receiving usual care, thereby reducing the number of prescription of medications with predicted drug-gene interactions (Oslin et al., 2022). In this study, we found no significant difference in the long-term effects of pharmacogenomics-guided antidepressant drug therapy on depressive symptoms, which is consistent with the results reported in most studies on depressive symptoms.

Interestingly, we found that pharmacogenomics-guided antidepressant treatment had a significant advantage in terms of reducing anhedonia and drug-related side effects in patients with first-time depression. It is widely known that anhedonia has been recognized as an early core symptom of depression and has been proven to be a predictive factor for poor outcomes among patients receiving antidepressant treatment (Bekhbat et al., 2022). Although epigenetic research on anhedonia is limited, it is hypothesized that the dopamine (DA) system is associated with the mechanism of dysfunction of the reward processing processing neural circuit in anhedonia (Sullivan et al., 2000). DA pathway-related gene polymorphisms may be risk genes for anhedonia, including catechol-O-methyltransferase (COMT), DA transporter 1 (DAT1), DA receptor D2 (DRD2), DRD3, DRD4, and monoamine oxidase A (MAOA) (Krach et al., 2010; Mier et al., 2010). On the basis of the N-methyl-Daspartate receptor (NMDAR) antagonist ketamine, which has a rapid relieving effect on depressive symptoms in MDD patients, some studies have proposed the glutamate hypothesis of anhedonia (Lee et al., 2013). Huang et al. (Xinxin et al., 2024) reported that the polymorphic interaction of glutamate pathway genes can affect the ability to experience pleasure, which may be a predictive factor for anhedonia. Moreover, adverse reactions may be a key predictive factors of the effectiveness of antidepressant treatment (Filip et al., 2024). A study involving a cohort of 251,745 depression patients revealed that 54% of respondents chose to stop taking antidepressant medication within 1 month after receiving a prescription, with medication side effects being the most common factor affecting compliance, with a reporting rate of 20%–40% (Hung, 2014). The screening of toxic genes through pharmacogenomics can reduce a risk of adverse reactions (Zhou et al., 2015). Previous studies have shown that depression patients with CC genotype have a significantly higher risk of developing nausea and vomiting after 4 weeks of treatment with citalopram than patient with the CT + TT genotype (Demirbugen Oz et al., 2018). The encoding of serotonin transporters and S/S (short) genotypes is believed to be associated with slower clearance of 5-HT in synaptic cleft, which may lead to increased sensitivity of the gastrointestinal tract to antidepressant drugs (Kato and Serretti, 2010). However, only one evidence-based study of antidepressant treatment guided by pharmacogenomics used adverse events as the primary outcome measure. The results revealed that the incidence of early drop-out associated with adverse events was also lower in pharmacogenomic-based antidepressant treatment group than in the control group, which is consistent with our findings (Han et al., 2018).

In the real world, there are differences in systematic evaluation, clinical diagnosis, and treatment experience among different seniority clinicians, which may have an impact on the final clinical outcome (Tsugawa et al., 2017). In a machine learning challenge, there was a difference of 7.3 months in bone age assessment of 316 children on bone radiographs among senior, mid-level, and junior radiologists compared with a difference of 4.3 months in the AI-assisted assessment (Wang et al., 2022). In particular, owing to the lack of objective evidence-based medical indicators, this method relies more on the subjective clinical experience of psychiatrists in the diagnosis and treatment of psychiatry (De Crescenzo et al., 2022). Our study revealed that there was no significant difference in the outcome of symptoms in various dimensions at the endpoint of treatment between depression patients with senior psychiatrists and depression patients with junior psychiatrists, which conflicts with our traditional beliefs. The Australian and New Zealand clinical practice guidelines for the treatment of depression suggest that antidepressant treatment should be guided by pharmacogenomic guidelines, but clinical practice still depends on the skills and preferences of psychiatrists (Ellis, 2004). Notably, highly experienced psychiatrists consider the burden and benefits of treatment, including side effects and toxicity, which can have a beneficial effect on the treatment outcomes (Benacek et al., 2024). This finding is similar to our conclusion that the frequency of antidepressant medication changes among junior psychiatrists is significantly higher than that among senior psychiatrists. The precise selection of drugs by senior psychiatrists may induce placebo effects in patients with depression, but to our knowledge, few studies have examined this phenomenon. Pharmacogenomics may be able to fill the gap in objective indicators for antidepressants selection.

Overall, pharmacogenomics may contribute to the clinical treatment of refractory depression or estimation of adverse drug reactions and has potential applications in epigenetic research. Compared with junior clinicians, experienced clinicians have a certain advantage in terms of the accuracy of antidepressant drug selection; however, there is a lack of evidence regarding their advantages in other dimensions. Pharmacogenomics may reduce the influence of clinicians’ experience on patient outcomes in primary healthcare settings.

### Limitations

Our research has many strengths, including a prospective study design, clinical practice challenges, acceptable sample size, long-term observation period, and multidimensional symptom and safety assessment. However, the limitations of our study should be noted. First, our findings should be validated in larger samples recruited from multiple centers. Second, clinical research cannot achieve complete double-blind significance. Additionally, clinical physicians’ knowledge regarding antidepressant may change over time. Future studies should increase the use of standardized clinical diagnosis and treatment procedures or cutting-edge precision testing tools for antidepressant treatment. These findings provide evidence-based support for the clinical application of psychiatrists in the treatment of depression.

## Conclusion

In summary, our fndings demonstrate that pharmacogenomics-guided antidepressant drug therapy can effectively mitigate the occurrence of anhedonia and side effects in patients with first-episode depression. Higher levels of clinical experience among psychiatrists can lead to significant reductions in the frequency of antidepressant drug changes, and the depressive symptoms at the endpoint are negatively correlated with clinicians’ work experience. Pharmacogenomics has important clinical significance in the treatment of depression, especially in primary mental healthcare settings, as it can reduce the influence of clinician experience.

## Data Availability

The original contributions presented in the study are included in the article/supplementary material, further inquiries can be directed to the corresponding authors.

## Abbreviations

5- HTT: 5-HT transporter; ANCOVA: Analysis of covariance; ANOVA: Analysis of variance; ASEC: Antidepressant Side Effect Checklist; BDNF: Brain-derived neurotrophic factor; BL: Baseline; BMI: Body mass index; COMT: Catechol-O-methyltransferase; CGI: Clinical Global Impression; CG, control group; DARS: Dimensional Anhedonia Rating Scale; DSM-5: Diagnostic and Statistical Manual of Mental Disorders, Fifth Edition; HPA: Hypothalamic pituitary adrenal axis; HAM-D17: 17-items Hamilton Depression Scale; LOCF: Last-observation-carrying-forward; MAOA: Monoamine oxidase A; MECT: Modified Electroconvulsive Therapy; MDD: Major depressive disorder; NMDAR: N-methyl-Daspartate receptor; PDQ-D: Perceived Deficits Questionnaire-Depression; PG: pharmacogenomic-guided group; PGx: Pharmacogenomics; PXR: Pregnane X receptor; QIDS-SR: Quick Inventory of Depressive Symptomatology Self Report; RM ANOVA: Repeated measures analysis of variance; RM MANOVA: Repeated measure multivariate analysis of variance; rTMS: Repetitive Transcranial Magnetic Stimulation; Wk, week.
